# Dual Identity Development and Adjustment in Muslim Minority Adolescents

**DOI:** 10.1007/s10964-019-01117-9

**Published:** 2019-09-13

**Authors:** Olivia Spiegler, Ralf Wölfer, Miles Hewstone

**Affiliations:** 1 Department of Experimental Psychology, University of Oxford, Woodstock Road, Oxford, OX2 6AE UK; 2 School of Psychology, University of Newcastle, Newcastle, Australia

**Keywords:** Dual identity, Ethnic minority adolescents, Muslim immigrants, Adjustment, Growth mixture models, Identity acculturation

## Abstract

Dual identity (e.g., strong ethnic and national identity) is a psychological resource for minority groups, but how it develops during adolescence is less clear. In this 3-wave longitudinal study, a person-oriented approach was used to examine dual identity development in a sample of 2145 Muslim adolescents (*M*_*T1*_ = 15 years, 51% female) in four Western European countries. The results of a growth-mixture model pointed toward four distinct developmental Classes: (1) “Dual identity”, (2) “Separation to dual identity”, (3) “Assimilation to dual identity”, and (4) “Separation”. Multiple group comparisons further showed that adolescents in Class 1 were well adjusted, but well-being (e.g., internalizing problems, life satisfaction) and health were even higher among adolescents in Class 2. Adolescents in Class 3 had consistently lower levels of well-being, and adolescents in Class 4 had lower levels of socio-cultural adjustment (e.g., problem behaviour at school, delinquent behaviour, and lack of intergroup contact). The findings underscore that most Muslim minority adolescents in Western Europe develop a dual identity, and that the developmental process, not simply the outcome, matters for adjustment.

## Introduction

Adolescence is a critical period for identity development during which adolescents become increasingly aware of how social group memberships impact their life chances, and gain new cognitive capacities that enable them to negotiate and explore multiple social identities in greater depth (Umaña-Taylor et al. 2014). For ethnic minority group adolescents this involves the development of an ethnic identity (e.g., a sense of belonging to the ethnic community and heritage country) and the development of a national identity (e.g., identification with the country in which adolescents grow up). These identities are dynamic, multidimensional psychological constructs and include, for example, adolescents’ sense of belonging and emotional attachments to groups (Phinney et al. 2001). The development of a dual identity (e.g., strong ethnic and national identity) is a central aspect of the acculturation process that is known to have beneficial effects for ethnic minority group members’ well-being and adjustment (Nguyen and Benet-Martinez 2013). Yet, little is known about the formation of dual identities (Amiot et al. 2018). In addition, there is growing evidence that identities can appear stable over time on a sample-averaged level whereas substantial fluctuation is found on an individual level (Huang and Stormshak 2011; Spiegler et al. 2018; Stoessel et al. 2014). This study aims to contribute to this literature by using a person-oriented approach, that uncovers classes of individuals with distinct developmental trajectories, as opposed to a variable-centred approach that identifies single growth trajectories (e.g., Jung and Wickrama 2008).

The study focuses on Muslim minority adolescents in Western Europe, a group that is of interest for at least three reasons. First, Muslims constitute a large and increasing, but understudied part of the immigrant population in Western societies (e.g., Europe, the U.S. or Canada). Second, Muslims are an at-risk population for identity-based threats as public debates are often driven by the fear that Muslims fail to integrate (Foner 2015), and because anti-Muslim sentiments are widespread (Kaya 2015). It is of great importance to investigate whether minority adolescents, if indeed they wish to, can develop dual identities in such challenging contexts. Third, dual identities are linked to better well-being and adjustment for members of many ethnic minority groups (Nguyen and Benet-Martínez 2013), but not necessarily for ethnic minority groups who face pervasive prejudice and more incompatible group identities (Baysu et al. 2011; Iyer et al. 2009). It is therefore important to study whether dual identities are a psychological resource or a source of distress for Muslim minority adolescents.

### Dual Identity Development

Dual identity is present when ethnic minority group members have both a strong ethnic and a strong national identity. However, there are other ways for ethnic minority group members to combine both identities. Identity acculturation models (Berry 1997; Phinney et al. 2001) propose, for example, that both identities can be weak, which indicates either marginalization or an individualization strategy. Minority group members may alternatively emphasize one identity over the other (e.g., prioritizing their ethnic or national identity, which indicates identity separation or assimilation, respectively). Prior research among Muslim adolescents (Kunst et al. 2012) and young adults (Fleischmann and Phalet 2018) in Western Europe points towards identity separation, but this research used cross-sectional data and a variable-centered approach, thereby neglecting the possibility of subgroups, each following a different developmental trajectory (e.g., dual identity development). Exceptions include work by Spiegler et al. (2018) who studied dual identity development in Turkish early adolescents, and Zhang et al. (2018) who studied dual identity in a cross-sectional sample of Muslim adults.

Longitudinal person-oriented research on dual identity development during adolescence is scarce, and there is none on Muslim minority adolescents. Moreover, the existing studies frequently uncover more than a single growth trajectory. Schwartz et al. (2015), for example, followed 302 recent-immigrant Hispanic adolescents (15 years at baseline) in the U.S. over a period of two years. On a sample-averaged level, they found no change in U.S. or ethnic identity. However, the results of a latent class growth analysis showed that there were two distinct classes of adolescents. While one of these groups (53%) mapped the sample-averaged picture of moderate and stable identities, the other group (47%) was characterized by strong and increasing ethnic and national identities. Knight et al. (2009) followed 332 mostly male Mexican American adolescent offenders (14–15 years at baseline) over the course of three years. One of their measures approximated adolescents’ identifications/affiliations (e.g., “I associate with Anglos”). On average, they found strong ethnic and moderate national identification/affiliation (both stable). However, by using a person-oriented approach they identified two distinct classes of adolescents: the first (62%) had moderately strong ethnic and national identities (both stable), while the second (38%) had strong ethnic and weak national identities. Finally, Stoessel et al. (2014) investigated 366 first-generation ethnic German diaspora immigrants in Germany (16 years at baseline) and identified three distinct classes. Adolescents in the largest class (46%) had strong, but decreasing ethnic identities and consistently weak national identities; those in the second largest class (28%) had weak, increasing ethnic identities and consistently strong national identities; and those in the smallest class (25%) had consistently moderate ethnic and national identities. Taken together, these findings point toward heterogeneity in dual identity development but also toward common developmental patterns across national contexts and minority groups. All three studies found, for example, a class with moderate dual identities, and two studies found an identity separation class in which ethnic identities were significantly stronger than national identities. A class with strong dual identities or identity assimilation, on the other hand, was found in only one study in each case. None of the studies found a class with marginalized identities.

### Dual Identity and Adjustment

Group identities are important psychological resources (Jetten et al. 2014), and linked to ethnic minority group members’ adjustment. The frequently examined adjustment domains in the acculturation literature include psychological adjustment, socio-cultural adjustment, and health related outcomes (Nguyen and Benet-Martinez 2013). Psychological adjustment refers to emotional well-being, and includes depression, self-esteem, internalizing problems, and life satisfaction. Socio-cultural adjustment refers to behavioural competencies, and includes social skills (e.g., friendships, peer acceptance), and behavioural problems (e.g., delinquent behaviour). Health related outcomes include, for example, somatic symptoms (e.g., headaches) and healthy behaviour (e.g., sleeping). Prior research indicates that strong ethnic identities are associated with better adjustment as they provide people with a sense of belonging, relatedness, and continuity. Meta-analytic evidence from the U.S., for example, found small to medium associations between ethnic identity and psychological adjustment, and small to medium associations between ethnic identity and socio-cultural adjustment (Rivas-Drake et al. 2014). National identities are another source of group belonging that can translate into well-being (Bobowik et al. 2017), academic success (Kiang and Witkow 2018), and more diverse friendship relations (Leszczensky 2018). While both identities are independently linked to positive outcomes, meta-analytic evidence suggests that the combination of them is most beneficial for ethnic minority group members’ psychological and sociocultural adjustment and to some extent also health-related outcomes (Nguyen and Benet-Martinez 2013). The various explanations for the advantage of dual identities over identification with just one group include the nurturing of creativity and flexibility, advanced cross-cultural competences, and extended social support networks (Nguyen and Benet-Martinez 2013). Taken together, ethnic and national identities, and especially dual identities, appear to be important psychological resources for ethnic minority group members’ adjustment.

Despite the overwhelming evidence for the adaptive advantage of dual identities, research has also highlighted that dual identity development can be a stressful and challenging task. Different normative expectations of groups, for example, can lead to the perception of incompatible identities which produces behavioural conflicts and uncertainty (e.g., Hirsh and Kang 2016). Dual identities also seem to be less beneficial in hostile and exclusionary contexts, where a focus on ingroup resources and comfort might be the more adaptive acculturation strategy (Baysu et al. 2011). Finally, dual identities might be adaptive in one domain but less so in others. Research among British-Asian children, for example, showed that dual identities were linked to better socio-cultural adjustment but lower psychological well-being (Brown et al. 2013). Taken together, these studies suggest that dual identities might be less beneficial in hostile and exclusionary contexts and when the two identities appear incompatible, both of which conditions apply in the case of Muslim minorities in Western societies (Hutchinson et al. 2015; Pew Research Centre 2016).

## Current Study

The aims of the present study were threefold. The first aim was to characterize dual identity development on a sample-averaged level. Based on previous studies among Muslim minority adolescents and early adults, a pattern of identity separation was expected. The second aim was to examine if there were classes of adolescents with distinct developmental trajectories. Identity acculturation theories and prior person-oriented research suggested that there should be at least three groups of adolescents: a group with dual identities, a group with separated identities, and a group with assimilated identities. The third aim was to explore whether Muslim adolescents’ dual identity development was longitudinally linked to a broad variety of developmental outcomes (e.g., psychological adjustment, socio-cultural adjustment, and health outcomes).

## Methods

### Procedure and Sample Description

Data were drawn from the Children of Immigrants Longitudinal Survey in Four European Countries (CILS4EU; Kalter et al. 2016a, 2016b, 2016c). Starting in 2010, there were three annual waves of measurement. Data were collected in England, Germany, the Netherlands, and Sweden. A total of 18,646 ethnic minority and majority students were recruited through a school-based sample selection design whereby schools with a high proportion of immigrant and immigrant-origin adolescents were oversampled. The participation rates were high (e.g., school participation = 84%, class participation within participating schools = 99%, and student participation within participating classes = 85%). At Waves 1 and 2 all students were interviewed in school, whereas at Wave 3 respondents were followed-up individually and interviewed via phone, mail, or web when they had left school or when schools did not want to take part in the survey again.

To address the research aims, all three waves of measurement were used and only first and second-generation adolescents who reported being Muslim were included (*n* = 2950). Because of the focus on dual identity, adolescents who did not feel that they belonged to at least one ethnic group in addition to the national group were excluded. Therefore, the final sample comprised *n* = 2145 adolescents, including 339 adolescents from England (47% female; *M*_*ageT1*_ = 15.08 years, *SD*_*ageT1*_ = 4.60 months), 736 from Germany (50% female, *M*_*ageT1*_ = 15.41 years, *SD*_*ageT1*_ = 8.87 months), 513 from the Netherlands (49% female, *M*_*ageT1*_ = 15.29 years, *SD*_*ageT1*_ = 7.87 months), and 557 from Sweden (57% female, *M*_*ageT1*_ = 14.67 years, *SD*_*ageT1*_ = 5.10 months). Adolescents in England originated primarily from Pakistan (61%), India (10%) and Bangladesh (8%). In Germany, adolescents’ country of origin was most frequently Turkey (73%), followed by Serbia (5%) and Lebanon (4%). In the Netherlands, adolescents originated from Turkey (42%) and Morocco (37%), and Iraq (4%). Adolescents in Sweden were most heterogenous in their country of origin: Iraq (16%), Bosnia and Herzegovina (13%), Somalia (12%), Kosovo-Albania (9%), Turkey (9%), Iran (6%), Lebanon (5%), and Kurdistan (5%). More information about the sample can be found in Table 1.

**Table 1:** Descriptive statistics

	Total (*n* = 2145)	England (*n* = 339)	Germany (*n* = 736)	Netherlands (*n* = 513)	Sweden (*n* = 557)	
Social background						
Female	51.1%	47.0%_a_	50.1%_a,b_	49.1%_a,b_	56.6%_b_	*F* (3,2140) = 3.32*
Age T1 in years	15.14 (0.67)	15.07 (0.38)_a_	15.41 (0.74)_b_	15.30 (0.66)_c_	14.67 (0.42)_d_	*F* (3,2088) = 167.61***
1st generation	23.0%	26.0%_a_	18.9%_b_	18.5%_b_	30.9%_a_	*F* (3,2141) = 11.50***
Father university	28.4%	36.5%_a_	11.5%_b_	18.9%_c_	57.0%_d_	*F* (3,1777) = 118.40***
Mother university	19.8%	17.6%_a_	5.8%_b_	11.8%_c_	48.4%_d_	*F* (3,1838) = 138.84***
Region of origin						
Asian-Pacific	19.2%	78.5%_a_	3.5%_b_	6.0%_b_	15.8%_c_	*F* (3,2141) = 563.99***
European	12.7%	3.0%_a_	11.8%_b_	2.7%_a_	29.1%_c_	*F* (3,2141) = 77.48***
Middle-East	22.0%	3.5%_a_	9.5%_b_	44.4%_c_	29.1%_d_	*F* (3,2141) = 116.52***
Sub-Sahara Africa	7.2%	9.4%_a_	2.6%_b_	2.5%_b_	16.3%_c_	*F* (3,2141) = 39.31***
Turkey	37.5%	1.2%_a_	72.6%_b_	42.1%_c_	9.0%_d_	*F* (3,2141) = 403.57***
Dual identity						
Ethnic identity T1	3.44 (0.67)	3.33 (0.69)_a_	3.47 (0.63)_b_	3.64 (0.56)_c_	3.28 (0.76)_a_	*F* (3,2081) = 29.68***
Ethnic identity T2	3.46 (0.67)	3.38 (0.62)_a_	3.50 (0.62)_b_	3.59 (0.61)_b_	3.36 (0.78)_a_	*F* (3,1424) = 8.66***
Ethnic identity T3	3.40 (0.68)	3.34 (0.63)_a,b_	3.49 (0.67)_b_	3.48 (0.64)_b_	3.25 (0.72)_a_	*F* (3842) = 6.83***
National identity T1	2.58 (0.89)	2.99 (0.77)_a_	2.24 (0.93)_b_	2.81 (0.86)_c_	2.55 (0.78)_d_	*F* (3,2116) = 77.81***
National identity T2	2.66 (0.89)	2.99 (0.77)_a_	2.42 (0.96)_b_	2.82 (0.86)_c_	2.66 (0.81)_d_	*F* (3,1647) = 32.65***
National identity T3	2.82 (0.84)	3.14 (0.71)_a_	2.63 (0.91)_b_	2.91 (0.75)_c_	2.84 (0.77)_c_	*F* (3,1116) = 18.35***
Adjustment						
Problem behaviour at school T1	1.78 (0.69)	2.03 (0.80)_a_	1.69 (0.59)_b_	1.89 (0.73)_c_	1.65 (0.67)_b_	*F* (3,2137) = 30.05***
Problem behaviour at school T2	1.71 (0.67)	1.81 (0.73)_a_	1.66 (0.60)_b_	1.77 (0.74)_a,b_	1.65 (0.65)_b_	*F* (3,1556) = 5.14**
Problem behaviour at school T3	1.70 (0.66)	1.74 (0.63)_a_	1.67 (0.61)_a,b_	1.53 (0.56)_b_	1.75 (0.75)_a_	*F* (3765) = 2.80*
Delinquent behaviour T1	0.33 (0.72)	0.35 (0.75)_a,b_	0.39 (0.73)_b_	0.25 (0.64)_a_	0.32 (0.75)_a,b_	*F* (3,1915) = 3.73*
Delinquent behaviour T2	0.31 (0.72)	0.27 (0.67)	0.32 (0.70)	0.36 (0.83)	0.30 (0.66)	*F* (3,1612) = 0.84
Delinquent behaviour T3	0.20 (0.56)	0.15 (0.44)	0.23 (0.58)	0.21 (0.67)	0.16 (0.51)	*F* (3,1115) = 1.65
Intergroup contact T1	3.36 (1.23)	2.64 (1.06)_a_	2.83 (1.27)_a_	2.27 (1.18)_b_	2.73 (1.22)_a_	*F* (3,2024) = 21.86***
Intergroup contact T2	3.51 (1.19)	2.38 (1.07)_a,c_	2.61 (1.19)_b_	2.33 (1.24)_a_	2.55 (1.21)_b,c_	*F* (3,1624) = 5.29**
Intergroup contact T3	3.32 (1.24)	2.42 (1.14)_a_	2.73 (1.27)_b_	2.59 (1.25)_a,b_	2.82 (1.22)_b_	*F* (3,1090) = 4.44**
Internalizing problems T1	1.93 (0.65)	2.15 (0.67)_a_	2.09 (0.65)_a_	1.81 (0.59)_b_	1.70 (0.60)_c_	*F* (3,2135) = 60.08***
Internalizing problems T2	2.01 (0.75)	2.03 (0.78)_a_	2.23 (0.72)_b_	1.87 (0.63)_c_	1.87 (0.80)_c_	*F* (3,1557) = 24.20***
Internalizing problems T3	2.04 (0.70)	2.15 (0.79)	2.01 (0.62)	–	2.03 (0.75)	*F* (3911) = 2.96
Life satisfaction T1	7.96 (2.09)	7.69 (2.05)_a_	7.47 (2.42)_a_	8.24 (1.65)_b_	8.53 (1.84)_b_	*F* (3,2116) = 33.14***
Life satisfaction T2	7.97 (2.21)	7.74 (2.04)_a_	7.61 (2.43)_a_	8.51 (1.59)_b_	8.16 (2.32)_b_	*F* (3,1644) = 14.68***
Life satisfaction T3	7.91 (1.80)	7.58 (1.92)_a_	8.06 (1.79)_b_	7.86 (1.68)_a,b_	7.90 (1.81)_a,b_	*F* (3,1109) = 3.14*
Health T1	3.46 (0.87)	3.35 (0.88)_a_	3.41 (0.85)_a,b_	3.51 (0.86)_b_	3.54 (0.88)_b_	*F* (3,2134) = 4.90**
Health T2	3.39 (0.91)	3.34 (0.91)	3.40 (0.88)	3.48 (0.92)	3.35 (0.94)	*F* (3,1628) = 1.65
Health T3	3.47 (0.87)	3.45 (0.85)_a,b_	3.55 (0.83)_b_	3.51 (0.80)_a,b_	3.33 (0.98)_a_	*F* (3,1097) = 3.87**

Means (standard deviations in parentheses). Equal subscript letters in a row denote similarity (*p* < 0.05). Degrees of freedom change due to missing data

*p < 0.05, **p < 0.01, ***p < 0.001

### Measures

#### Dual identity

National and ethnic identity (T1–T3) were measured with one item each, capturing respondents’ feeling of belonging to the country in which they live and the ethnic community. Adolescents were first asked to indicate the strength of their national identity, “How strongly do you feel [survey country member]?” on a scale that ranged from 1 (*not at all strongly)* to 4 (*very strongly)*. In a next step, respondents’ sense of ethnic identity was assessed by asking them to indicate whether they felt that they belong to other groups as well. They were presented with a list of groups and asked to tick all that apply. The list always included the group linked to their country of origin (e.g., “Turkey” for adolescents of Turkish heritage). However, ethnic options such as “Kurdistan”, “Berber”, “Kosovo-Albania” or “Chechen” were also included. Respondents then used the same 4-point scale to respond to the following item: “How strongly do you feel that you belong to this group? (If you feel you belong to more than one of these groups, please tell us about the one you feel you belong to most strongly.)”.

#### Adjustment

In the CILS4EU study, various adjustment scales were available, and all those that could be identified as an indicator of one of the adjustment domains (e.g., psychological, socio-cultural, or health) and were longitudinally available (T1–T3) were included. For example, self-esteem was excluded from the analyses as it was only available for T1. Intergroup contact, in contrast, was included as an indicator of socio-cultural adjustment since it requires behavioural competencies and social skills to form and maintain (intimate) cross-cultural relations. Taken together, there were three indicators of socio-cultural adjustment (i.e., problem behaviour at school, delinquent behaviour, and intergroup contact), two measures of psychological adjustment (i.e., life-satisfaction, internalizing problems), and one physical well-being measure (i.e., health).

##### Problem behaviour at school

Problem behaviour at school refers to disruptive and unacceptable student behaviour and was measured with four items, rated on a 5-point scale from 1 *(never)* to 5 (*every day*): “How often do you answer back to your teachers?”, “How often do you get a punishment at school (such as being sent out of class, writing lines, getting a detention)?”, “How often do you skip a lesson without permission?”, “How often do you arrive late at school?”. Cronbach’s alphas were 0.71, 0.70, and 0.68 (T1–T3, respectively).

##### Delinquent behaviour

Delinquent behaviour refers to criminal, anti-social, and offending behaviour of adolescents. It was measured with four items: “Have you done the following things in the past 3 months? Deliberately damaged things that were not yours?”, “Stolen something from a shop/from someone else?”, “Carried a knife or weapon?”, and “Been very drunk?”. To encourage disclosure, respondents were reminded that their answers would be kept secret. The response options were 0 (*no*) or 1 (*yes*). Sum scores were calculated, so that delinquent behaviour ranged from 0 (*no delinquent behaviour*) to 4 (*high delinquent behaviour*).

##### Intergroup contact

Intergroup contact was operationalized as cross-group friendships which refers to positive, intimate, and enduring relationships with majority group peers. Contact was measured with one item: “Thinking about all of your friends. How many of them have a [White British/Dutch/Swedish/German] background?”. Response options were 1 (*none of or very few*), 2 (*a few*), 3 (*about half*), 4 (*a lot*), and 5 (*almost all or all*).

##### Internalizing problems

Internalizing problems refer to adolescents’ emotional and psychological well-being. It includes negative affect and emotions such as feeling depressed or anxious. Internalizing problems were measured with four items: “I feel anxious/very worried/depressed/worthless.”. The scale ranged from 1 (*never true*) to 4 (*often true*). Cronbach’s alphas (T1–T3) were 0.76, 0.86, and 0.75.

##### Life satisfaction

Life satisfaction refers to the degree to which a person positively evaluates the overall quality of their life as a whole. It was measured with a single item: “On a scale from 1 to 10 where 1 is very unsatisfied and 10 is very satisfied, how satisfied are you with your life in general?”

##### Health

Health refers to adolescents’ physical well-being and was measured with three items rated on a 5-point scale from 1 (*every day*) to 5 (*never*): “In the last six months, how often have you had a headache/a stomach ache/difficulties falling asleep?”. Cronbach’s alphas (T1–T3) were 0.68, 0.71, and 0.68.

#### Demographic variables

At Wave 1 adolescents reported on their gender, age, immigrant generation, mother’s and father’s education (i.e., University degree vs. no University degree), and region of origin (i.e., Asian-Pacific, European, Middle-East, Sub-Sahara Africa, and Turkey).

### Measurement Invariance

As recommended for cross-cultural research (Byrne and van de Vijver 2017), alignment optimization was used to determine the degree of approximate measurement invariance across the four countries for multiple item scales. At least 75% of the parameters (e.g., intercepts and loadings) should be invariant to achieve approximate measurement invariance (Muthén and Asparouhov 2014). The results showed that 92% of the parameters were invariant across countries at Time 1, 90% at Time 2, and 95% at Time 3. The measures can therefore be considered invariant (see OSM Table 1 for details).

### Missing Data

Wave 2 data were obtained from 1665 adolescents (78%); these remaining respondents differed only marginally from adolescents who dropped out (*d* < 0.20; with two exceptions: adolescents who dropped out were slightly older and had slightly more problem behaviour at school, *d* = 0.22 and *d* = 0.25, respectively). At Wave 3, 1131 adolescents continued to participate (62%), and remaining respondents differed again marginally from adolescents who dropped out (*d* < 0.20; with two exceptions: boys and adolescents with fewer internalizing problems were more likely to drop out, *d* = 0.22 and *d* = 0.31, respectively). Therefore, attrition can be considered mostly unsystematic (see OSM Table 2 for details). Non-responses ranged from 0% (generational status) to 17% (father education) at Wave 1, from 0.8% (national identity) to 6.3% (problem behaviour at school) at Wave 2, and from 0.4% (national identity) to 32.5% (problem behaviour at school) at Wave 3. However, problem behaviour at school was not assessed among respondents who had left school. Full information maximum likelihood estimation was used to handle missing data.

**Table 2:** Correlations Time 1 international sample

	Sex	Age	GEN	EDF	EDM	EI	NI	PBS	DEL	CON	INP	SAT
Age	−0.10***											
GEN	−0.04	0.09***										
EDUF	−0.01	−0.19***	0.14***									
EDUM	−0.00	−0.19***	0.10***	0.54***								
EI	−0.02	0.06**	−0.02	−0.12***	−0.10***							
NI	0.02	−0.04	−0.07**	−0.00	−0.03	−0.08**						
PBS	−0.13***	0.07**	−0.01	−0.03	−0.03	0.01	−0.06**					
DEL	−0.17***	0.06**	−0.01	0.00	0.01	0.02	−0.13***	0.38***				
CON	−0.00	−0.02	0.03	0.05*	0.08**	−0.14***	0.15	−0.12***	−0.03			
INP	0.23***	0.12***	0.00	−0.09***	−0.12***	−0.14***	−0.06**	0.14***	0.12***	0.07**		
SAT	−0.16***	−0.10***	−0.02	0.10***	0.11***	0.12***	0.08***	−0.15***	−0.15***	−0.02	−0.47***	
HEA	−0.24***	−0.06*	0.04*	0.04	0.05*	0.08***	0.02	−0.23***	−0.15***	−0.01	−0.44***	0.27***

Sex (1 = boy and 2 = girl)

*GEN* generational status (1 = 1st, 0 = 2nd), *EDUF and EDUM* University degree father and mother (1 = university degree, 0 = not), *EI* ethnic identity, *NI* national identity, *PBS* problem behaviour at school, *DEL* delinquent behaviour, *CON* intergroup contact, *INP* internalizing problems, *SAT* life satisfaction, *HEA* health

*p < 0.05, **p < 0.01, ***p < 0.001

### Statistical Analyses

In line with the three study aims, analyses were conducted in three steps. First, a parallel process latent growth curve model (LGCM) was specified to gain an overall impression of dual identity development. This variable-centred approach resulted in a single growth trajectory for ethnic and national identity. Second, a parallel process growth mixture model (GMM) was specified. This person-oriented approach uncovered classes of adolescents who followed different development trajectories. Third, differences between the classes of dual identity development in terms of adjustment were examined. To do so, a LGCM for each adjustment variable was specified and multiple-group analyses were used to test whether the levels and changes in adjustment varied by class. The analyses were conducted in MPlus 7.4 (Muthén and Muthén 1998–2015). The estimator MLR (maximum likelihood estimation with robust standard errors) and TYPE = COMPLEX (when applicable) were used to account for non-normality, stratification, and non-independence of observations.

## Results

### Average Dual Identity Development

The descriptive statistics are shown in Tables 1–3. First, a parallel process LGCM was specified based on the longitudinal measures of ethnic and national identity. This model estimated intercepts and slopes for both identities, which can be interpreted as an adolescent’s initial level and rate of change over time. Technically, this was achieved by fixing the time scores of the slope factors at 0, 1, and 2. The residual variance of the T1 manifest ethnic identity variable was fixed at zero. The slopes were correlated and regressed on the intercepts of the other identity.

**Table 3:** Correlations Time 2 (below diagonal) and Time 3 (above diagonal) international sample

	1	2	3	4	5	6	7	8
Ethnic identity	–	−0.13***	−0.06	0.03	−0.15***	−0.06	0.15***	0.04
National identity	−0.11***	–	−0.11**	−0.04	0.22***	−0.05	0.07	0.01
Problem behaviour at school	−0.04	−0.09**	–	0.25 ***	−0.10**	0.23***	−0.22***	−0.26***
Delinquent behaviour	−0.00	−0.10***	0.41***	–	−0.01	0.04	−0.14***	−0.08*
Intergroup contact	−0.13***	0.16***	−0.05*	−0.03	–	0.03	−0.00	−0.05
Internalizing problems	−0.12***	−0.05*	0.14***	0.07*	0.07**	–	−0.52***	−0.50***
Life satisfaction	0.15***	0.02	−0.17***	−0.12***	−0.02	−0.52***	–	0.31***
Health	0.08**	0.03	−0.25***	−0.13***	−0.01	−0.44***	0.26***	–

*p < 0.05, **p < 0.01, ***p < 0.001

**Fig. 1:**
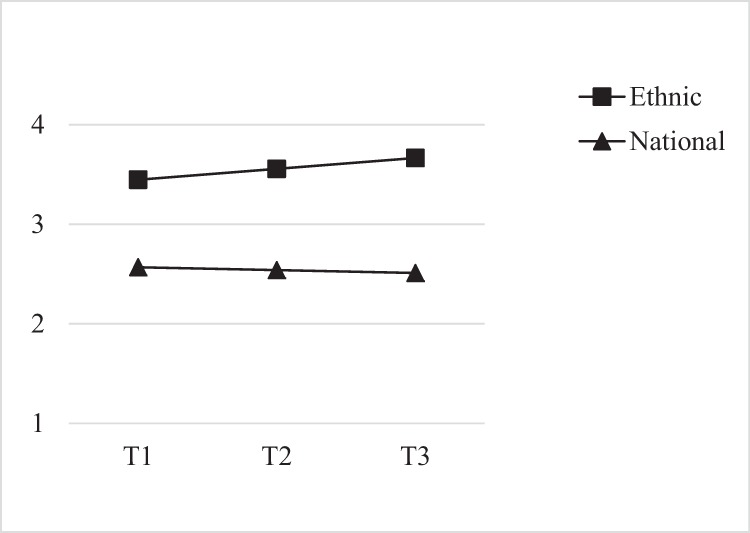
Average dual identity development

The model fit the data well: χ^2^(df) = 19.56 (9), TLI = 0.983, CFI = 0.990, RMSEA = 0.023, 90% CI (0.008, 0.038), and the results indicated that adolescents had on average relatively strong and stable ethnic identities *b(SE)* = 3.45 (0.01), *p* < 0.001, *m(SE)* = 0.11 (0.08), *p* = 0.155, and moderately strong and stable national identities *b(SE)* = 2.57 (0.02), *p* < 0.001, *m(SE)* = −0.03 (0.22), *p* = 0.896 (Fig. 1). To examine whether the levels and slopes were significantly different from each other, the fit of a constrained model in which they were fixed to be the same was compared to the fit of an unconstrained model in which they could differ. If the constrained model fit significantly worse, this indicated that intercepts and slopes were different. As MLR was used, the Satorra-Bentler scaling correction was used to adjust χ^2^. The results indicated that ethnic identity was significantly stronger than national identity, χ^2^(1) = 795.44, *p* < 0.001, whereas the rates of change were similar, χ^2^(1) = 0.44, *p* = 0.509. To examine how both identities influenced each other over time, the associations between the intercepts and slopes were inspected. Stronger initial levels of ethnic identity were linked to weaker initial levels of national identity *r* = −0.05, *p* < 0.001; all other associations were non-significant, *p*s ≥ 0.120.

### Classes of Dual Identity Development

The second aim was to identify classes of dual identity development. Therefore, GMM was applied in which growth parameter means are freely estimated and allowed to differ across a set of classes, so that classes can have different intercepts and slopes. The variance and covariance of the growth parameters were freely estimated but held equal across classes, so that within each class individuals could vary around the class-specific intercept and slope, but across classes the variation was equal.

To identify the appropriate number of classes, an unconditional, parallel process GMM that included two classes was specified. A stepwise procedure was used, whereby one additional class (k) was added to the model at a time and the fit of the more parsimonious model compared with the model with one additional class. All models were estimated with a sufficient number of random starts to achieve a replicated log-likelihood (LL) value. To decide on the number of classes, the Bayesian Information Criterion (BIC) was used which should be lower when compared to the k−1 class solution. In addition, the Lo–Mendell–Rubin Likelihood Ratio Test (LMR–LRT) and the Bootstrapped Likelihood Ratio Test (BLRT) were used. These tests evaluate the adequacy of a k−1 class solution compared to a k-class solution, whereby a significant difference indicates that the k-class solution fits the data better. Solutions in which classes contained 5% of the total sample or less were not considered. Finally, parsimony and theoretical meaning of the classes were considered. The model fit statistics of the class solutions are presented in Table 4. The LMR-LRT pointed toward a four-class solution, and the BIC values and the BLRT results toward a five-class solution. However, one of the classes in the five-class solution contained only 1% of the total sample, thus a four-class solution was preferable.

**Table 4:** Model fit statistics, GMM analyses and class sizes

Classes	BIC	LMR–LRT	BLRT	Entropy	n_1_	n_2_	n_3_	n_4_	n_5_
2	20,446.25	−10,322.38	−10,322.38***	0.941	1149	995			
3	16,526.32	−10,131.08	−10,131.08***	0.979	827	1142	176		
4	16,509.61	−8151.94**	−8151.94***	0.839	827	830	176	311	
5	16,508.91	−8124.41	−8124.41***	0.851	827	821	176	302	18

Class sizes are reported based on the estimated posterior probabilities. Higher-class solutions were inadmissible

**p < 0.01, ***p < 0.001

**Fig. 2:**
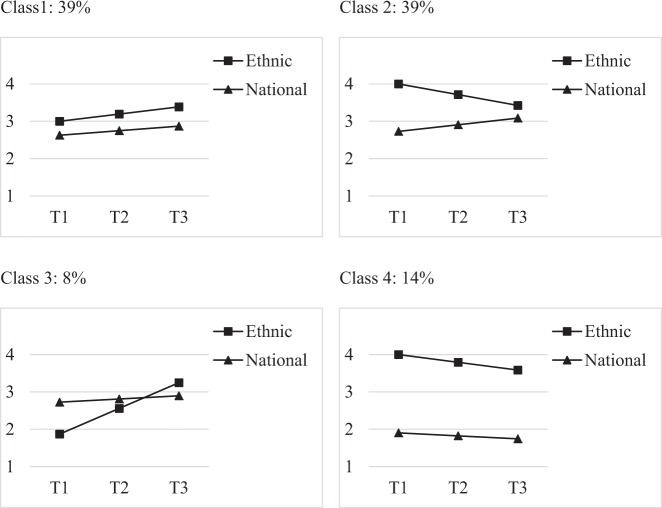
Subgroups of dual identity development

Figure 2 shows the four distinct developmental paths of dual identity development. Adolescents in Class 1 (39%) had moderately strong and increasing ethnic and national identities, *b(SE)* = 3.00 (0.00), *m(SE)* = 0.19 (0.02), and *b(SE)* = 2.63 (0.03), *m(SE)* = 0.12 (0.02), respectively, all *p*s < 0.001. Adolescents in Class 2 (39%) had strong ethnic identities that decreased over time, *b(SE)* = 4.00 (0.00), *m(SE)* = −0.29 (0.02), while their moderately strong national identities increased, *b(SE)* = 2.73 (0.08), *m(SE)* = 0.18 (0.03), all *p*s < 0.001. Adolescents in Class 3 (8%) had weak but sharply increasing ethnic identities, *b*(*SE*) = 1.87 (0.03), *m*(*SE*) = 0.69 (0.05), *p*s < 0.001, and moderately strong national identities, that slightly increased, *b*(*SE*) = 2.73 (0.06), *p* < 0.001, *m*(*SE*) = 0.09 (0.04), *p* = 0.025. Adolescents in Class 4 (14%) had strong ethnic identities that decreased over time, *b*(*SE*) = 4.00 (0.00), *m*(*SE*) = −0.21 (0.02), *p*s < 0.001, and continuously weak national identities *b*(*SE*) = 1.90 (0.11), *p* < 0.001, *m*(*SE*) = −0.08 (0.11), *p* = 0.480.

The ethnic identity levels of the four classes were significantly different from each other (*p*s < 0.001), except for Classes 2 and 4, Wald χ^2^(1) = 1.27, *p* = 0.259. All ethnic identity slopes differed from each other (*p*s < 0.008). National identity levels were similar for Classes 1, 2, and 3 (*p*s ≥ 0.130), but significantly weaker in Class 4 (*p*s < 0.001). The differences between the national identity slopes just failed to reach significance, for example, Class 2 vs. 3 Wald χ^2^(1) = 3.48, *df* = 1, *p* = 0.062, Class 2 vs. 4, Wald χ^2^(1) = 3.42, *df* = 1, *p* = 0.064, and Class 1 vs. 4 Wald χ^2^(1) = 3.01, *df* = 1, *p* = 0.083.

For descriptive purposes only, class differences in terms of demographic characteristics including country of settlement and region of origin were explored. A detailed description of the findings can be found online (OSM Table 3). Gender and generational status were not related to class membership, but younger adolescents and those with parents educated at a higher level were more likely in Class 3. With respect to country of settlement, findings indicated that Dutch adolescents were more likely in Class 2, Swedish adolescents were more likely in in Class 3, and German adolescents more likely in Class 4, where English adolescents were least likely. With respect to region of origin, findings indicated that adolescents from the Middle East were more likely in Class 2, those from Asian pacific and Sub-Saharan African countries in Class 3, and those from Turkey in Class 4. Originating from a European country was not related to class membership. The GMM analysis was repeated within each country of settlement. This replicated many, but not all, classes, indicating country specific patterns of dual identity development (see OSM Tables 4–6, and Fig. 1).

**Table 5:** Model fit statistics for unconstrained multiple group models

	χ^2^ (df)	*p* for χ^2^	TLI	CFI	RMSEA	90% C.I.
Problem behaviour at school	3.09 (7)	0.877	1.021	1.000	0.000	0.000, 0.027
Delinquent behaviour	14.73 (7)	0.040	0.921	0.954	0.046	0.010, 0.080
Intergroup contact	4.15 (6)	0.656	1.007	1.000	0.000	0.000, 0.046
Internalizing problems	9.24 (7)	0.236	0.992	0.996	0.025	0.000, 0.062
Life satisfaction	7.13 (7)	0.415	0.999	0.999	0.006	0.000, 0.054
Health	3.17 (6)	0.787	1.009	1.000	0.000	0.000, 0.037

To improve the model fit for intergroup contact and health, we freely estimated the intercepts of the T2 manifest variables

**Table 6:** Results for multiple group comparisons

		Class 1	Class 2	Class 3	Class 4
Problem behaviour at school	Intercept	1.76 (0.03)_a_	1.79 (0.03)_a_	1.78 (0.06)_a_	1.81 (0.04)_a_
	Slope	−0.03 (0.03)_a,b_	−0.10 (0.03)**_a_	−0.03 (0.08)_a,b_	0.05 (0.07)_b_
Delinquent behaviour	Intercept	0.33 (0.03)_a_	0.30 (0.03)_a_	0.34 (0.05)_a,b_	0.44 (0.05)_b_
	Slope	−0.11 (0.03)**_a_	−0.13 (0.03)***_a_	−0.12 (0.05)*_a_	−0.16 (0.05)**_a_
Intergroup contact	Intercept	2.75 (0.05)_a_	2.52 (0.05)_b_	2.99 (0.10)_c_	2.44 (0.09)_b_
	Slope	−0.03 (0.06)_a_	0.17 (0.06)**_b_	−0.09 (0.12)_a_	−0.16 (0.09)_a_
Internalizing problems	Intercept	2.00 (0.03)_a_	1.82 (0.02)_b_	2.14 (0.06)_c_	1.94 (0.04)_a_
	Slope	0.07 (0.03)*_a_	0.08 (0.03)**_a_	0.10 (0.07)_a_	0.09 (0.05)_a_
Life satisfaction	Intercept	7.75 (0.08)_a,c_	8.29 (0.07)_b_	7.42 (0.19)_a_	7.91 (0.14)_c_
	Slope	−0.00 (0.09)_a_	−0.02 (0.09)_a_	0.04 (0.22)_a_	0.12 (0.15)_a_
Health	Intercept	3.39 (0.03)_a_	3.53 (0.03)_b_	3.32 (0.07)_a_	3.51 (0.05)_b_
	Slope	0.05 (0.04)_a_	0.04 (0.04)_a_	0.10 (0.08)_a_	0.02 (0.05)_a_

Unstandardized effects (standard errors in parentheses). χ^2^ difference tests (*df* = *1, p* ≤ 0.05) were conducted for each pair of classes and adjusted using the Satorra-Bentler scaling correction. Different subscripts in a row indicate differences between classes

*p < 0.05, **p < 0.01, ***p < 0.001

### Classes of Dual Identity Development and Adjustment

To explore the associations between class membership and adjustment, the longitudinal measures of problem behaviour at school, delinquent behaviour, intergroup contact, internalizing problems, life satisfaction, and physical well-being were used to build six latent growth curve models. The factor loadings were fixed to 0 for the T1 manifest variables, freely estimated for the T2 manifest variables, and fixed to 1 for the T3 manifest variables. As a result, the slope estimates referred to a change between the first and third wave. For identification purposes the residual variances of the T1 manifest variables were fixed at 0. For delinquent behaviour the residual variance of the T3 manifest variable was fixed at 0.

Multiple group comparisons were used to examine if the structural parameters (e.g., intercepts and slopes) differed between classes. Specifically, the fit of a constrained model in which parameters (e.g., the intercepts of two classes) were fixed to be the same was compared to an unconstrained model in which the parameters could differ. The structural parameters varied between two classes if the constrained model fit significantly worse than the unconstrained model. The magnitude of the difference between models was estimated with the *ω* effect size, which accounts for sample size and degrees of freedom, and can be interpreted with reference to standard conventions of small (*ω* = 0.1), medium (*ω* = 0.3), and large (*ω* = 0.5) effect sizes (Newsom 2015; but see Funder and Ozer 2019, for a critical review of these benchmarks).

The model fit statistics for the unconstrained multiple group models are shown in Table 5, and the results of the multiple group comparisons in Table 6. Problem behaviour at school did not differ across the four classes, but the decline in Class 2 differed significantly from the (non-significant) increase in Class 4 [χ^2^(1) = 4.19, *p* = 0.041, *ω* = 0.05]. Delinquent behaviour declined across the four classes but was consistently higher in Class 4 compared to Class 1 [χ^2^(1) = 6.01, *p* = 0.014, *ω* = 0.05], and Class 2 [χ^2^(1) = 14.31, *p* < 0.001, *ω* = 0.08]. Adolescents in Class 3 had more intergroup contact than adolescents in Class 1 [χ^2^(1) = 5.32, *p* = 0.021, *ω* = 0.05], Class 2 [χ^2^(1) = 23.99, *p* < 0.001, *ω* = 0.11], and Class 4 [χ^2^(1) = 18.48, *p* < 0.001, *ω* = 0.09]. In addition, adolescents in Class 1 had more intergroup contact than adolescents in Class 2 [χ^2^(1) = 15.09, *p* < 0.001, *ω* = 0.09] and 4 [χ^2^(1) = 9.65, *p* = 0.002, *ω* = 0.07]. The increase in intergroup contact in Class 2 differed significantly from the (non-significant) decrease in Class 1 [χ^2^(1) = 8.13, *p* = 0.004, *ω* = 0.06], Class 3 [χ^2^(1) = 4.35 *p* = 0.037, *ω* = 0.05], and Class 4 [χ^2^(1) = 9.43, *p* = 0.002, *ω* = 0.07]. Internalizing problems were consistently higher in Class 3 compared to Class 1 [χ^2^(1) = 5.54, *p* = 0.019, *ω* = 0.05], Class 2 [χ^2^(1) = 33.45, *p* < 0.001, *ω* = 0.13], and Class 4 [χ^2^(1) = 8.69, *p* = 0.003, *ω* = 0.06]. In addition, internalizing problems were lower in Class 2 compared to Class 1 [χ^2^(1) = 33.59, *p* < 0.001, *ω* = 0.13], and Class 4 [χ^2^(1) = 8.27, *p* = 0.004, *ω* = 0.06]. Life satisfaction was consistently lower in Class 3 compared to Class 2 [χ^2^(1) = 30.89, *p* < 0.001, *ω* = 0.12], and Class 4 [χ^2^(1) = 4.52, *p* = 0.034, *ω* = 0.05]. In addition, life satisfaction was higher in Class 2 compared to Class 1 [χ^2^(1) = 27.24, *p* < 0.001, *ω* = 0.11], and Class 4 [χ^2^(1) _C2C4_ = 7.15, *p* = 0.007, *ω* = 0.06]. Health was consistently lower in Class 1 compared to Class 2 [χ^2^(1) = 10.74, *p* = 0.001, *ω* = 0.07], and Class 4 [χ^2^(1) = 3.86, *p* = 0.049, *ω* = 0.04], and health was consistently lower in Class 3 compared to Class 2 [χ^2^(1) = 7.52, *p* = 0.006, *ω* = 0.06], and Class 4 [χ^2^(1) = 4.79, *p* = 0.029, *ω* = 0.05]. As a robustness check these analyses were repeated a) without data imputation (i.e., using only respondents with complete information from T1–T3), and b) with various combinations of covariates such as gender, generational status, and region of origin. The results are similar and can be found in the online Supplementary materials (Tables 7 and 12).

## Discussion

Dual identities are a psychological resource for ethnic minorities, but the development of dual identities is not well understood. Therefore, this study examined changes in ethnic and national identity during adolescence. On a sample averaged level, moderately separated identities were found and no change over the course of two years. However, the use of a person-oriented approach uncovered four groups of adolescents with distinct developmental trajectories, and only one, relatively small group had separated identities. Adolescents in the other three groups had dual identities or were in the process of developing such an identity. The study further showed that identity development was systematically linked to developmental outcomes (i.e., psychological, socio-cultural, and health outcomes). The following paragraphs consider, first, what this study contributes to the field of dual identity development, and, second, how dual identity development is related to adjustment among Muslim minority adolescents. Finally, strengths, limitations, and directions for future research are outlined.

### Dual Identity Development

This research shows that Muslim minority adolescents follow different developmental trajectories, rather than a single normative pattern, when it comes to the negotiation of their dual belonging. A relatively large group of adolescents (Class 1) had a dual identity. This interpretation is in line with prior research arguing that a dual identity does not require a *very* strong sense of belonging (Simon and Ruhs 2008). Another large group of adolescents (Class 2) had separated identities at the onset of the study but developed towards a dual identity over time. One could argue that these adolescents were assimilating due to their decreasing ethnic and increasing national identities. Assimilation is, however, conceptualized as distance from the ethnic ingroup (e.g., Berry 1997; Phinney et al. 2001) which did not apply because ethnic identities remained strong despite the decline. The smallest group of adolescents (Class 3) came closest to what could be cautiously described as assimilation, because ethnic identities were weaker than national identities at the onset of the study. Adolescents in this group also developed a dual identity over time. Finally, there was a relatively small group of adolescents (Class 4) with consistently separated identities. The label separation was ascribed to this group despite the somewhat decreasing ethnic identities, and because of the consistently large gap between both identities. A group of adolescents with marginalized identities was not found, which is in line with prior research (Knight et al. 2009; Schwartz et al. 2015; Stoessel et al. 2014).

While the classes can broadly be mapped onto identity acculturation theories (e.g., Berry 1997; Phinney et al. 2001), the findings also extend these theories in important ways. The results highlight, for example, that dual identities are likely to be moderately strong in populations that face identity-based threats. The longitudinal perspective further indicates that assimilation may be primarily a transitory state for stigmatized adolescents. Finally, more flexible and longitudinal views on identity acculturation are needed. It seems, for example, that dual identity and separation can be characterized by ethnic identity declines (on a very high level).

This study further adds to the literature on ethnic-racial identity development in at least two ways. First, considerable developmental variability over time was uncovered. Previous longitudinal studies indicated that ethnic identities are stable during late adolescence (Birman and Trickett 2001; Kiang et al. 2013; Pahl and Way 2006). On a sample-averaged level, the findings accord with this view. However, on an individual level, ethnic identities were not stable – they increased, sharply increased, or decreased. This pattern highlights that older adolescents are still actively engaged in a dynamic process of ethnic identity development. Second, adolescents’ national identity was included as an important related aspect of the self with implications for minority group members’ adjustment (Berry 1997; Phinney et al. 2001).

### Dual Identity Development and Adjustment

The findings showed that Muslim minority adolescents in Western Europe, like many other ethnic minority groups (Nguyen and Benet-Martinez 2013), benefit from dual identities. However, it seems to be the *process* of developing a dual identity, rather than the outcome itself, that matters. This becomes evident when comparing Classes 1 to 3. While adolescents in all three classes had moderate dual identities at the end of the study, Class 2 was better adjusted than Classes 1 and 3 in terms of psychological well-being, socio-cultural adjustment, and health. Consequently, it is not just having a dual identity that is linked to better adjustment, but the various ways of getting there. Based on this study, one could argue that the most adaptive trajectory for Muslim minority adolescents is to have a very strong ethnic identity at the end of middle adolescence (as a resource for psychological well-being), and to become more dually identified during late adolescence.

The findings further showed that a persistent lack of positive intergroup contact is linked to continued identity separation and weak national identities. Higher, or increasing, levels of intergroup contact, in contrast, were related to gradual increases in national identity, and this did not happen at the expense of losing one’s ethnic identity. The link between intergroup contact and national identity growth is in line with cross-sectional research among Muslim minority adolescents (Fleischmann and Phalet 2018). Relatedly, the intergroup contact literature shows that enduring, intimate contact with majority group members reduces intergroup anxiety, perceived inequalities, and group differences which, in turn, inspire a sense of social acceptance and shared belonging among minorities (Brown and Hewstone 2005), in particular during adolescence (Wölfer et al. 2016).

It appeared that identity separation was linked to less sociocultural competence (e.g., more delinquent behaviour, less intergroup contact), whereas initial assimilation was mostly linked to reduced well-being (e.g., more internalizing problems, lower life satisfaction) and health. This indicates that assimilation is psychologically more demanding than separation. A possible explanation is offered by research within the acculturation gap framework. This work shows that assimilated adolescents are more engaged with the host culture and less engaged with the heritage culture than their parents, which can lead to family conflicts, parent-child disengagement, and poorer family functioning (Costigan and Dokis 2006); all of these factors decrease adolescents’ psychological well-being, but not (or less so) their ability to function in majority group contexts (Schwartz et al. 2013).

The finding that separated adolescents experienced more social difficulties accords with previous findings among sojourners (Ward and Kennedy 1994), and might be explained by the expectations of majority group members toward ethnic minorities. Minority group members are expected to interact with majority group members, to be loyal to the majority group and country, and to obtain cultural knowledge to negotiate daily social interactions (Rohmann et al. 2006). A lack of national identity, however, indicates difficulties in achieving this goal (Ward and Kennedy 1994). This can become a hassle for individuals who are expected to blend in, and more antisocial behaviours may be a response to these difficulties.

It is worth mentioning that the effects of class membership on adjustment were relatively small, with the amount of explained variance ranging from 4 to 13%. This aligns perfectly with prior meta-analytic evidence showing that biculturalism explains 10% of the variance in adjustment in ethnic minority samples outside the US (Nguyen and Benet-Martinez 2013), and research among Muslim minority adults in Western Europe showing that dual identity explains 3 to 14% of the variance of psychological well-being indicators (Zhang et al. 2018). Moreover, we found the largest effects of class membership? for adolescents’ internalizing problems and life satisfaction, and comparatively small effects for problem behaviour at school and health. This indicates that dual identities are primarily a protective resource for Muslim minority adolescents’ psychological well-being, and of secondary importance for their behavioural adjustment and health.

### Strengths, Limitations, and Future Research Directions

The strengths of this research include its longitudinal design with a large, international sample. Longitudinal designs allow one to better understand developmental processes, and large international data sets help mitigate against context- and sample specific findings. Another strength is the focus on an understudied minority population. Up to date there is only limited research on Muslim minority adolescents in Western Europe, which is in sharp contrast to the public interest in this population. Understanding how these adolescents relate to their heritage and the societies in which they grow up is vital for an informed discussion of integration. Finally, rigorous analytical methods (e.g., LGCM, GMM) were used to shed light on the heterogeneity within this population and to approximate the number of adolescents who psychologically separate from the societies in which they grow up.

The following limitations, which signal directions for future research, need to be acknowledged. A first limitation refers to the person-oriented approach (e.g., GMM). Like other clustering procedures, GMM results are sample specific which is why they need to be replicated in future research with other samples. A second limitation might be that GMM are not the preferred method to study temporal relations. While these were not the focus of the research, prior work suggests that dual identity development predicts adjustment (e.g., Benish-Weisman et al. 2015) and intergroup friendships (Leszczensky 2018). Third, only single item measures were used to capture multifaceted and complex constructs (e.g., social identity, intergroup contact). More comprehensive measures are desirable, but for reasons of cost and survey completion time, population-based data often cannot accommodate more extensive item sets for all constructs. In the case of identification, there is at least evidence for the validity of single item measures (Postmes et al. 2013) which increases confidence in these specific findings. Fourth, various important aspects of adjustment were included, such as psychological well-being, socio-cultural adjustment, and health. Nevertheless, it would have been interesting to include academic achievement as dual identities might not be beneficial for this adjustment domain, especially when captured with objective measures such as school grades (Schotte et al. 2018) or school track (Baysu et al. 2011). However, these measures were not consistently available across countries and time. Fifth, the study relied on self-reports which can inflate observed associations between variables. Future research with multiple informants and more objective measures of adjustment should, therefore, provide a stronger test for the findings. Finally, the study cannot provide a comprehensive view on dual identity development during adolescence as the data covered only three waves and two years. More large scale, longitudinal research with a focus on intragroup heterogeneity is needed to uncover where these classes come from and how they continue to develop.

## Conclusion

Dual identities have a vital impact on ethnic minority group members’ psychological and socio-cultural adjustment, but still relatively little is known about their development during adolescence and whether findings thus far also apply to highly stigmatized groups. This study therefore focused on dual identity development among Muslim minority adolescents in Western Europe. The findings, which draw attention to distinct developmental paths that differed substantially from the sample-averaged picture of stable identity separation, have at least three implications. First, a focus on intragroup variability is important and should be considered in addition to the well-established methodological approaches in the field. Second, minority adolescents continue to develop their identities well into late adolescence, which should inform research on ethnic-racial identity development. Third, most Muslim minority adolescents in Western Europe have or develop moderate dual identities which is relevant for public debates claiming that Muslims fail to integrate; they do integrate, in the sense of having a valued national identity, but they also cherish an ethnic identity, which in no sense precludes either a willingness or an ability to integrate. Finally, dual identities can be a psychological resource for highly stigmatized minority adolescents; however, it is the process of getting there that matters. Muslim adolescents in Western Europe appear to benefit most from very strong ethnic identities early in adolescence and a gradual engagement with the national group during late adolescence.

## Supplementary Information

Supplementary Information
