# Supplementary material for: Dual Identity Development and Adjustment in Muslim Minority Adolescents
Source: J Youth Adolesc. 2019 Sep 13;48(10):1924–37. doi: 10.1007/s10964-019-01117-9 (PMC6813286; doi:10.1007/s10964-019-01117-9)
Supplement: Supplementary file 1 — Supplementary Information [file 10964_2019_1117_MOESM1_ESM.docx]

*Table 1.* Alignment results. Approximate measurement (non) invariance for intercepts and loadings across countries

|  |  | Wave 1 |  | Wave 2 |  | Wave 3 |  |
| --- | --- | --- | --- | --- | --- | --- | --- |
| Factor | Item | Loadings | Intercepts | Loadings | Intercepts | Loadings | Intercepts |
| Problem behavior school | argue with teacher | 1 2 3 (4) | 1 2 (3) (4) | 1 2 3 (4) | (1) 2 3 4 | 1 2 3 4 | 1 2 3 4 |
|  | punishment | 1 2 3 (4) | 1 2 3 (4) | 1 2 3 (4) | 1 (2) 3 (4) | 1 2 3 4 | 1 2 3 4 |
|  | skip lesson | 1 2 3 4 | 1 2 3 4 | 1 2 3 4 | 1 2 3 4 | 1 2 3 4 | 1 2 3 4 |
|  | late to school | 1 2 3 4 | 1 2 3 4 | 1 2 3 4 | 1 2 3 4 | 1 2 3 4 | 1 2 3 4 |
| Delinquency | damaged things | 1 2 3 4 | (1) 2 3 4 | 1 2 3 4 | (1) 2 3 4 | 1 2 3 4 | 1 2 3 4 |
|  | stolen | 1 2 3 4 | (1) 2 3 4 | 1 2 3 4 | (1) 2 3 4 | 1 2 3 4 | 1 2 3 4 |
|  | knife or weapon | 1 2 3 4 | 1 2 3 4 | 1 2 3 4 | 1 2 3 4 | 1 2 3 4 | 1 2 3 4 |
|  | very drunk | 1 2 3 4 | 1 2 3 4 | 1 2 3 4 | 1 2 3 4 | 1 2 3 4 | 1 2 3 4 |
| Internalizing problems | worried | 1 2 3 4 | 1 (2) (3) 4 | 1 2 3 4 | 1 (2) (3) 4 | 1 2 3 4 | 1 (2) (3) 4 |
|  | anxious | 1 2 3 4 | (1) (2) 3 4 | 1 2 3 (4) | 1 (2) 3 (4) | 1 2 3 4 | 1 (2) (3) 4 |
|  | depressed | 1 2 3 4 | 1 2 3 4 | 1 2 3 4 | 1 (2) 3 4 | 1 (2) 3 4 | 1 2 3 4 |
|  | worthless | 1 2 3 4 | 1 2 3 4 | 1 2 3 4 | 1 2 3 4 | 1 2 3 4 | 1 (2) (3) 4 |
| Health | headache | 1 2 3 4 | 1 2 3 4 | 1 2 3 4 | 1 2 3 4 | 1 2 3 4 | 1 2 3 4 |
|  | stomach ache | 1 2 3 4 | 1 2 3 4 | 1 2 3 4 | 1 2 3 4 | 1 2 3 4 | 1 2 3 4 |
|  | sleep difficulties | 1 2 3 4 | 1 2 3 4 | 1 2 3 4 | 1 2 3 4 | 1 2 3 4 | 1 2 3 4 |

*Notes:* Numbers indicate the country (1 = England, 2 = Germany, 3 = Netherlands, 4 = Sweden). The parentheses indicate whether the parameter (intercept or factor loading) is non-invariant for that specific country.

*Table 2.* Detailed drop-out analyses based on international sample

|  | Wave 2 | | | Wave 3 | | |
| --- | --- | --- | --- | --- | --- | --- |
|  | Participants  (*n* = 1,665) | Drop out  (*n* = 480) | Effect size  (Cohen’s *d*) | Participants  (*n* = 1,033) | Drop out  (*n* = 632) | Effect size  (Cohen’s *d*) |
| Age | 15.11 (.66) | 15.26 (.70) | **.22** | 15.10 (.66) | 15.13 (.65) | .05 |
| Sex | 1.51 (.50) | 1.51 (.50) | .00 | 1.55 (.50) | 1.44 (.50) | **.22** |
| Generational status | 0.23 (.42) | 0.25 (.43) | .05 | 0.22 (.42) | 0.23 (.42) | .02 |
| University degree father | 0.29 (.45) | 0.28 (.45) | .02 | 0.26 (.44) | 0.34 (.47) | .18 |
| University degree mother | 0.20 (.40) | 0.19 (.39) | .03 | 0.19 (.40) | 0.21 (41) | .05 |
| Ethnic identity | 3.42 (.68) | 3.51 (.63) | .13 | 3.39 (.69) | 3.47 (.68) | .12 |
| National identity | 2.59 (.88) | 2.54 (.95) | .06 | 2.57 (.88) | 2.62 (.87) | .06 |
| Majority group friends | 3.36 (1.21) | 3.38 (1.27) | .02 | 3.30 (1.12) | 3.44 (1.20) | .12 |
| Problem behaviour at school | 1.74 (.67) | 1.92 (.76) | **.25** | 1.71 (.63) | 1.78 (.72) | .10 |
| Delinquent behaviour | 0.30 (.69) | 0.43 (.82) | .17 | 0.29 (.65) | 0.32 (.73) | .04 |
| Internalizing problems | 1.94 (.66) | 1.92 (.63) | .03 | 2.01 (.67) | 1.81 (.63) | **.31** |
| Satisfaction with life | 7.95 (2.12) | 7.99 (2.00) | .02 | 7.80 (2.14) | 8.21 (2.07) | .19 |
| Health | 3.46 (.86) | 3.44 (.89) | .02 | 3.41 (.85) | 3.55 (.88) | .16 |

*Notes*. Means (standard deviations in parentheses). Effect sizes contrast participants versus drop outs on Wave 1 variables. Coding: sex (1 = boy, 2 = girl), generational status (1 = 1^st^, 0 = 2^nd^), university degree (1 = yes, 0 = no), living with both biological parents (0 = yes, 1 = no).

*Table 3.* Results of analyses of variance indicating differences between classes on study variables

|  | Class 1 | Class 2 | Class 3 | Class 4 |  |
| --- | --- | --- | --- | --- | --- |
| Social background |  |  |  |  |  |
| Sex | 1.51 (.50) | 1.50 (.50) | 1.54 (.50) | 1.49 (.50) | *F*(3,2109) = 0.38 |
| Age T1 in years | 15.14 (.67) _a_ | 15.16 (.65) _a_ | 14.99 (.62) _b_ | 15.19 (.76) _a_ | *F*(3,2057) = 3.40 * |
| Generational status | 0.24 (.43) | 0.22 (.41) | 0.23 (.42) | 0.23 (.42) | *F*(3,2110) = 0.61 |
| Father university | 0.30 (.46) _a_ | 0.24 (.43) _a_ | 0.47 (.50) _b_ | 0.28 (.45) _a_ | *F*(3,1758) = 10.31 *** |
| Mother university | 0.21 (.41) _a_ | 0.17 (.37) _a_ | 0.34 (.47) _b_ | 0.19 (.39) _a_ | *F*(3,1813) = 7.64 *** |
| Region of origin |  |  |  |  |  |
| Asian-Pacific | 0.22 (.41) _a_ | 0.18 (.39) _a_ | 0.29 (.45) _b_ | 0.11 (.31) _c_ | *F*(3,2110) = 8.58 *** |
| European | 0.13 (.34) | 0.14 (.35) | 0.10 (.30) | 0.11 (.31) | *F*(3,2110) = .97 |
| Middle-East | 0.19 (.39) _a_ | 0.26 (.44) _b_ | 0.22 (.41) _a,b_ | 0.20 (.40) _a,b_ | *F*(3,2110) = 4.49 ** |
| Sub-Saharan Africa | 0.09 (.28) _a,b_ | 0.05 (.22) _a_ | 0.13 (.33) _b_ | 0.08 (.27) _a,b_ | *F*(3,2110) = 5.54 ** |
| Turkey | 0.38 (.48) _a_ | 0.37 (.48) _a_ | 0.27 (.45) _b_ | 0.50 (.50) _c_ | *F*(3,2110) = 8.84 *** |
| Country of settlement |  |  |  |  |  |
| England | 0.19 (.39) _a_ | 0.15 (.35) _a_ | 0.17 (.40) _a_ | 0.06 (.24) _b_ | *F*(3,2110) = 8.44 *** |
| Germany | 0.36 (.48) _a_ | 0.29 (.45) _a_ | 0.27 (.45) _a_ | 0.56 (.50) _b_ | *F*(3,2110) = 24.74 *** |
| Netherlands | 0.18 (.38) _a_ | 0.34 (.47) _b_ | 0.10 (.30) _c_ | 0.18 (.39) _a_ | *F*(3,2110) = 30.71 *** |
| Sweden | 0.28 (.45) _a_ | 0.23 (.42) _a,b_ | 0.46 (.50) _c_ | 0.19 (.40) _b_ | *F*(3,2110) = 16.25 *** |
| Adjustment |  |  |  |  |  |
| Problem behaviour at school T1 | 1.76 (.67) | 1.79 (.71) | 1.78 (.74) | 1.81 (.68) | *F*(3,2106) = 0.44 |
| Problem behaviour at school T2 | 1.72 (.68) | 1.68 (.62) | 1.71 (.69) | 1.77 (.78) | *F*(3,1537) = 0.95 |
| Problem behaviour at school T3 | 1.68 (.63) | 1.67 (.68) | 1.71 (.70) | 1.82 (.71) | *F*(3,757) = 1.27 |
| Delinquent behaviour T1 | 0.31 (.69) | 0.32 (.71) | 0.33 (.66) | 0.42 (.84) | *F*(3,1886) = 1.44 |
| Delinquent behaviour T2 | 0.34 (.74) _a,b_ | 0.25 (.63) _a_ | 0.30 (.64) _a,b_ | 0.45 (.94) _b_ | *F*(3,1592) = 4.01 ** |
| Delinquent behaviour T3 | 0.30 (.57) | 0.17 (.52) | 0.21 (.50) | 0.22 (.59) | *F*(3,1106) = 0.44 |
| Majority group friends T1 | 2.76 (1.22) _a_ | 2.52 (1.98) _b_ | 2.99 (1.20) _c_ | 2.43 (1.26) _b_ | *F*(3,1993) = 12.38 *** |
| Majority group friends T2 | 2.55 (1.16) _a_ | 2.47 (1.20) _a_ | 2.71 (1.23) _a_ | 2.23 (1.17) _b_ | *F*(3,1605) = 5.51 ** |
| Majority group friends T3 | 2.71 (1.24) _a_ | 2.74 (1.20) _a_ | 2.95 (1.29) _a_ | 2.26 (1.20) _b_ | *F*(3,1081) = 7.58 *** |
| Internalizing problem behaviour T1 | 2.00 (.65) _a_ | 1.82 (.62) _b_ | 2.14 (.71) _c_ | 1.94 (.67) _a_ | *F*(3,2104) = 17.01 *** |
| Internalizing problem behaviour T2 | 2.08 (.77) _a_ | 1.89 (.69) _b_ | 2.27 (.81) _c_ | 2.03 (.76) _a,b_ | *F*(3,1538) = 12.97 *** |
| Internalizing problem behaviour T3 | 2.07 (.69) _a,b_ | 1.94 (.69) _a_ | 2.22 (.72) _b_ | 2.06 (.70) _a,b_ | *F*(3,905) = 4.20 ** |
| Satisfaction with life T1 | 7.75 (1.99) _a,b_ | 8.29 (2.03) _c_ | 7.42 (2.36) _a_ | 7.91 (2.24) _b_ | *F*(3,2085) = 13.75 *** |
| Satisfaction with life T2 | 7.71 (2.27) _a_ | 8.38 (1.94) _b_ | 7.25 (2.40) _c_ | 7.96 (2.40) _a,b_ | *F*(3,1625) = 15.98 *** |
| Satisfaction with life T3 | 7.74 (1.77) _a,b_ | 8.13 (1.71) _b_ | 7.64 (1.93) _a_ | 8.03 (1.97) _a,b_ | *F*(3,1100) = 4.27 ** |
| Health T1 | 3.39 (0.87) _a,b_ | 3.53 (0.89) _b_ | 3.32 (0.83) _a_ | 3.51 (0.84) _b_ | *F*(3,2103) = 5.41 ** |
| Health T2 | 3.33 (0.90) _a,b_ | 3.48 (0.89) _b_ | 3.24 (0.90) _a_ | 3.42 (0.97) _a,b_ | *F*(3,1609) = 4.34 ** |
| Health T3 | 3.40 (0.88) | 3.56 (0.85) | 3.43 (0.94) | 3.47 (0.87) | *F*(3,1088) = 2.29 |

*Notes.* Means (standard deviations in parentheses). Equal subscript letters in a row denote similarity. Sex (1 = boy, 2 = girl), generational status (1 = 1^st^, 0 = 2^nd^), university degree (1 = yes, 0 = no), parents separated (0 = yes, 1 = no), region of origin / country of settlement (1 = yes, 0 = no).

* *p* < .05, ** *p* < .01, *** *p* < .001.

*Table 4.* Model fit statistics for LGCM analyses for each country of settlement

|  | χ^2^(df) | *p* for χ^2^ | TLI | CFI | RMSEA | 90% C.I. |
| --- | --- | --- | --- | --- | --- | --- |
| England | 13.43 (7) | 0.062 | 0.925 | 0.965 | .053 | .000, .096 |
| Germany | 4.43 (7) | 0.729 | 1.020 | 1.000 | .000 | .000, .033 |
| Netherlands | 2.24 (7) | 0.945 | 1.059 | 1.000 | .000 | .000, .008 |
| Sweden | 11.62 (7) | 0.114 | 0.952 | 0.978 | .035 | .000, .068 |

*Note.* LGCM denotes latent growth curve model.

*Table 5.* Model fit statistics GMM analyses and class sizes for each country of settlement

| Country | Classes | BIC | LMR–LRT | BLRT | Entropy | n_1_ | n_2_ | n_3_ | n_4_ |
| --- | --- | --- | --- | --- | --- | --- | --- | --- | --- |
| England | 2 | 3093.24 | -1504.05 *** | -1504.05 *** | 0.874 | 284 | 40 |  |  |
|  | 3 | 2667.03 | -1477.25 | -1477.25 *** | 0.982 | 155 | 140 | 30 |  |
|  | 4 | 2664.43 | -1249.69 | -1249.69 *** | 0.941 | 155 | 117 | 29 | 22 |
| Germany | 2 | 7349.76 | -3733.14 | -3733.14 *** | 0.977 | 338 | 398 |  |  |
|  | 3 | 5753.08 | -3595.67 | -3595.67 *** | 0.986 | 292 | 398 | 46 |  |
| Netherlands | 2 | 3790.69 | -2051.81 | -2051.81 *** | 0.975 | 161 | 341 |  |  |
| Sweden | 2 | 5434.09 | -2674.22 *** | -2674.22 *** | 0.838 | 467 | 85 |  |  |
|  | 3 | 4704.63 | -2644.44 | -2644.44 *** | 0.970 | 226 | 246 | 80 |  |
|  | 4 | 4718.21 | -2263.92 | -2263.92 | 0.858 | 226 | 170 | 80 | 76 |

*Notes.* GMM denotes growth mixture model. Inadmissible solutions or solutions with small classes (≤ 5 %) not reported. Class sizes based on estimated posterior probabilities.

* *p* < .05, ** *p* < .01, *** *p* < .001.

*Table 6*. Results of LGCM and GMM analyses for each country of settlement and previous class assignments

| Country | Model | Class | Previous class  (international analyses) | Ethnic identity | | National identity | |
| --- | --- | --- | --- | --- | --- | --- | --- |
|  |  |  |  | Intercept b(SE) | Slope m(SE) | Intercept b(SE) | Slope m(SE) |
| England | LGCM | - | - | 3.33 (0.03) *** | 0.01 (0.03) | 2.98 (0.05) *** | 0.07 (0.03) * |
|  | GMM | 1 | Class 1 | 3.00 (0.00) *** | 0.22 (0.03) *** | 2.92 (0.06) *** | 0.11 (0.03) ** |
|  |  | 2 | Class 2 | 4.00 (0.00) *** | -0.36 (0.04) *** | 3.25 (0.07) *** | 0.02 (0.07) |
|  |  | 3 | Class 3 | 1.79 (0.08) *** | 0.69 (0.11) *** | 3.15 (0.09) *** | 0.01 (0.07) |
|  |  | 4 | Class 4 | 4.00 (0.00) *** | -0.31 (0.13) * | 1.78 (0.43) *** | 0.05 (0.31) |
| Germany | LGCM | - | - | 3.48 (0.02) *** | 0.01 (0.02) | 2.34 (0.04) *** | 0.18 (0.03) *** |
|  | GMM | 1 | Class 1 | 3.00 (0.00) *** | 0.24 (0.03) *** | 2.36 (0.05) *** | 0.17 (0.04) *** |
|  |  | 2 | Class 2, Class 4 | 4.00 (0.00) *** | -0.24 (0.02) *** | 2.15 (0.05) *** | 0.19 (0.03) *** |
|  |  | 3 | Class 3 | 1.91 (0.04) *** | 0.75 (0.08) *** | 2.26 (0.14) *** | 0.14 (0.08) |
| Netherlands | LGCM | - | - | 3.65 (0.03) *** | -0.07 (0.03) ** | 2.18 (0.04) *** | 0.04 (0.03) |
|  | GMM | 1 | Class 1, Class 3 | 2.89 (0.03) *** | 0.28 (0.04) *** | 2.93 (0.06) *** | 0.04 (0.04) |
|  |  | 2 | Class 2, Class 4 | 4.00 (0.00) *** | -0.24 (0.03) *** | 2.76 (0.05) *** | 0.04 (0.04) |
| Sweden | LGCM | - | - | 3.30 (0.03) *** | -0.01 (0.03) | 2.54 (0.04) *** | 0.13 (0.02) *** |
|  | GMM | 1 | Class 1 | 3.00 (0.00) *** | 0.11 (0.03) ** | 2.60 (0.05) *** | 0.10 (0.04) ** |
|  |  | 2 | Class 2, Class 4 | 4.00 (0.00) *** | -0.29 (0.03) *** | 2.43 (0.05) *** | 0.15 (0.04) *** |
|  |  | 3 | Class 3 | 1.87 (0.04) *** | 0.59 (0.08) *** | 2.76 (0.08) *** | 0.09 (0.06) |

*Note.* LGCM and GMM denote latent growth curve model and growth mixture model, respectively.

* *p* < .05, ** *p* < .01, *** *p* < .001.

|  | Average | Class 1 | Class 2 | Class 3 | Class 4 |
| --- | --- | --- | --- | --- | --- |
| ENG |  |  |  |  |  |
|  | 100% | 48% | 36% | 9% | 7% |
| GER |  |  |  |  |  |
|  | 100% | 40% | 54% | 6% |  |
| NL |  |  |  |  |  |
|  | 100% | 32% | 68% |  |  |
| SW |  |  |  |  |  |
|  | 100% | 41% | 44% | 15% |  |

*Figure 1.* Average dual identity development and classes of dual identity development in each country. ■ ethnic identity ▲ national identity. ENG, GER, NL, SW denote England, Germany, The Netherlands, Sweden, respectively.

*Table 7.* Model fit statistics for unconstrained multiple group models for respondents with complete information at T1-T3.

|  | χ^2^(df) | *p* for χ^2^ | TLI | CFI | RMSEA | 90% C.I. |
| --- | --- | --- | --- | --- | --- | --- |
| Problem behaviour at school | 5.05 (7) | .654 | 1.012 | 1.000 | .000 | .000, .078 |
| Delinquent behaviour | 10.24 (7) | .176 | 0.956 | 0.974 | .046 | .000, .102 |
| Intergroup contact | 3.30 (6) | .770 | 1.012 | 1.000 | .000 | .000, .058 |
| Internalizing problems | 6.55 (6) | .364 | 0.997 | 0.999 | .022 | .000, .098 |
| Life satisfaction | 3.46 (7) | .840 | 1.043 | 1.000 | .000 | .000, .045 |
| Health | 4.70 (6) | .583 | 1.005 | 1.000 | .000 | .000, .072 |

*Notes.* For intergroup contact, internalizing problems, and health, we freely estimated the intercepts of the T2 manifest variables.

*Table 8*. Results for multiple group comparisons for respondents with complete information at T1-T3.

|  |  | Class 1 | Class 2 | Class 3 | Class 4 |
| --- | --- | --- | --- | --- | --- |
| Problem behaviour at school | Intercept | 1.67 (.04) _a_ | 1.65 (.04) _a_ | 1.68 (.09) _a_ | 1.70 (.08) _a_ |
|  | Slope | -0.02 (.04) _a_ | -0.03 (.04) _a_ | -0.06 (.09) _a_ | 0.04 (.06) _a_ |
| Delinquent behaviour | Intercept | 0.30 (.03) _a_ | 0.25 (.03) _a_ | 0.29 (.07) _a_ | 0.33 (.07) _a_ |
|  | Slope | -0.11 (.04) ** _a_ | -0.10 (.03) ** _a_ | -0.14 (.06) * _a_ | -0.12 (05) * _a_ |
| Intergroup contact | Intercept | 2.76 (.07) _a_ | 2.60 (.07) _a_ | 3.06 (.13) _b_ | 2.57 (.12) _a_ |
|  | Slope | -0.02 (.07) _a_ | 0.12 (.07) ^+^ _a_ | -0.10 (.14) _a,b_ | -0.29 (.10) ** _b_ |
| Internalizing problems | Intercept | 2.06 (.04) _a,b_ | 1.95 (.04) _a_ | 2.20 (.08) _b_ | 2.08 (.08) _a,b_ |
|  | Slope | -0.01 (.04) _a_ | -0.01 (.05) _a_ | 0.07 (.09) _a_ | -0.02 (.08) _a_ |
| Life satisfaction | Intercept | 7.63 (.11) _a_ | 8.14 (.11) _b_ | 7.39 (.24) _a_ | 7.62 (.20) _a_ |
|  | Slope | 0.12 (.10) _a_ | 0.04 (.13) _a_ | 0.18 (.27) _a_ | 0.38 (.19) * _a_ |
| Health | Intercept | 3.34 (.04) _a_ | 3.57 (.05) _b_ | 3.22 (.10) _a_ | 3.40 (.07) _a_ |
|  | Slope | 0.08 (.05) ^+^ _a_ | 0.01 (.04) _a_ | 0.21 (.10) * _a_ | 0.09 (.06) _a_ |

*Notes.* Unstandardized effects (standard errors in parentheses). χ^2^ difference tests (*df = 1, p* ≤ .05) were conducted for each pair of classes and adjusted using the Satorra-Bentler scaling correction. Different subscripts in a row indicate differences between classes at *p* ≤ .05. Sample size problem behaviour at school: *n* *=* 658 [Class 1: *n* = 282; Class 2: *n* = 238; Class 3: *n* = 60; Class 4: *n* = 78], delinquent behaviour: *n* *=* 882 [Class 1: *n* = 361; Class 2: *n* = 328; Class 3: *n* = 78; Class 4: *n* = 115], intergroup contact: *n* *=* 925 [Class 1: *n* = 390; Class 2: *n* = 326; Class 3: *n* = 82; Class 4: *n* = 127], internalizing problems: *n* *=* 766 [Class 1: *n* = 331; Class 2: *n* = 258; Class 3: *n* = 75; Class 4: *n* = 102], life satisfaction: *n* *=* 994 [Class 1: *n* = 409 Class 2: *n* = 359; Class 3: *n* = 92; Class 4: *n* = 134], and health: *n* *=* 976 [Class 1: *n* = 403, Class 2: *n* = 353; Class 3: *n* = 89; Class 4: *n* = 131]. There were no significant differences between the classes in terms of problem behaviour at school and delinquent behaviour. Intergroup contact was higher in Class 3 compared to Class 1 [χ^2^(1) = 4.76, *p* = .029, *ω* = .07], Class 2 [χ^2^(1) = 10.56, *p* = .001, *ω* = .11], and Class 4 [χ^2^(1) = 8.44, *p* = .004, *ω* = .10], and intergroup contact was higher in Class 1 compared to Class 2 at *p* ≤ .10 [χ^2^(1) = 3.01, *p* = .083, *ω* = .06]. Intergroup contact decreased more strongly in Class 4 compared to Class 1 [χ^2^(1) = 5.40, *p* = .020, *ω* = .08], and Class 2 [χ^2^(1) = 13.43, *p* < .001, *ω* = .12]. Internalizing problems were higher in Class 3 compared to Class 2 [χ^2^(1) = 7.76, *p* = .005, *ω* = .10]. Life satisfaction was higher in Class 2 compared to Class 1 [χ^2^(1) = 10.45, *p* = .001, *ω* = .10], Class 3 [χ^2^(1) = 10.85, *p* = .001, *ω* = .10], and Class 4 [χ^2^(1) = 6.18, *p* = .013, *ω* = .08]. Health was higher in Class 2 compared to Class 1 [χ^2^(1) = 15.05, *p* < .001, *ω* = .12], Class 3 [χ^2^(1) = 10.21, *p* = .001, *ω* = .10], and Class 4 [χ^2^(1) = 4.92, *p* = .027, *ω* = .07], and health increases more strongly in Class 3 compared to Class 2 at *p* ≤ .10 [χ^2^(1) = 3.84, *p* = .050, *ω* = .06].

^+^ *p* < .10, * *p* < .05, ** *p* < .01, *** *p* < .001.

*Table 9.* Model fit statistics for unconstrained multiple group models with gender, age, generational status, and parent education as covariates

|  | χ^2^(df) | *p* for χ^2^ | TLI | CFI | RMSEA | 90% C.I. |
| --- | --- | --- | --- | --- | --- | --- |
| Problem behaviour at school | 27.70 (27) | .247 | 0.997 | 0.999 | .007 | .000, .035 |
| Delinquent behaviour | 41.94 (27) | .033 | 0.908 | 0.965 | .032 | .009, .051 |
| Intergroup contact | 30.59 (26) | .244 | 0.983 | 0.994 | .018 | .000, .041 |
| Internalizing problems | 44.11 (27) | .020 | 0.947 | 0.980 | .035 | .014, .053 |
| Life satisfaction | 21.37 (20) | .376 | 0.987 | 0.997 | .011 | .000, .040 |
| Health | 11.24 (26) | .995 | 1.041 | 1.000 | .000 | .000, .000 |

*Notes.* For intergroup contact and health, we freely estimated the intercepts of the T2 manifest variables. For internalizing problems, we fixed the residual variance of the T3 (instead of T1) manifest variable at 0. For life satisfaction, the residual variance of the T1 manifest variable was freely estimated, and father university degree correlated with T3 life satisfaction. Covariates include gender, age, generational status, university degree mother, and university degree father. Covariates were allowed to correlate.

*Table 10*. Results for multiple group comparisons with gender, age, generational status, and parent education as covariates

|  |  | Class 1 | Class 2 | Class 3 | Class 4 |
| --- | --- | --- | --- | --- | --- |
| Problem behaviour at school | Intercept | 1.96 (.09) _a_ | 2.09 (.09) _a_ | 2.18 (.20) _a_ | 2.22 (.13) _a_ |
|  | Slope | -0.05 (.11) _a_ | -0.03 (.10) _a_ | -0.38 (.22) _a_ | -0.09 (.17) _a_ |
| Delinquent behaviour | Intercept | 0.66 (.09) _a_ | 0.71 (.08) _a_ | 0.43 (.18) _a_ | 1.06 (.16) _b_ |
|  | Slope | -0.10 (.13) _a_ | -0.25 (.12) * _a_ | 0.05 (.22) _a_ | -0.30 (.18) _a_ |
| Intergroup contact | Intercept | 2.68 (.08) _a_ | 2.47 (.08) _b_ | 2.93 (18) _a_ | 2.52 (.15) _a,b_ |
|  | Slope | 0.08 (.08) _a_ | 0.13 (.09) _a_ | 0.04 (.24) _a,b_ | -0.33 (.15) * _b_ |
| Internalizing problems | Intercept | 1.60 (.07) _a_ | 1.48 (.06) _a,b_ | 1.79 (.17) _a_ | 1.28 (.11) _b_ |
|  | Slope | -0.09 (.11) _a,b_ | -0.09 (.12) _b_ | -0.59 (.23) * _a_ | 0.04 (.18) _b_ |
| Life satisfaction | Intercept | 8.55 (.21) _a_ | 9.29 (.22) _b_ | 8.24 (.60) _a,b_ | 9.16 (.36) _a,b_ |
|  | Slope | 0.11 (.16) _a_ | -0.33 (.18) _a_ | 1.13 (.47) * _b_ | -0.37 (.31) _a_ |
| Health | Intercept | 4.02 (.10) _a_ | 4.10 (.10) _a_ | 3.74 (.23) _a_ | 4.22 (.16) _a_ |
|  | Slope | 0.32 (.12) ** _a_ | 0.24 (.12) * _a_ | 0.68 (.25) ** _a_ | 0.15 (.17) _a_ |

*Notes.* Unstandardized effects (standard errors in parentheses). χ^2^ difference tests (*df = 1, p* ≤ .05) were conducted for each pair of classes and adjusted using the Satorra-Bentler scaling correction. Different subscripts in a row indicate differences between classes at *p* ≤ .05. Covariates are listed under Table 9. Problem behaviour at school was higher in Class 4 compared to Class 1 at *p* ≤ .10 [χ^2^(1) = 2.97, *p* = .085, *ω* = .04]. Delinquent behaviour was higher in Class 4 compared to Class 1 [χ^2^(1) = 5.41, *p* = .020, *ω* = .05], Class 2 [χ^2^(1) = 4.92, *p* = .027, *ω* = .05], and Class 3 [χ^2^(1) = 4.94, *p* = .026, *ω* = .05]. Intergroup contact was higher in Class 3 compared to Class 2 [χ^2^(1) = 5.06, *p* = .025, *ω* = .05], and Class 4 at *p* ≤ .10 [χ^2^(1) = 3.09, *p* = .079, *ω* = .04], and intergroup contact was higher in Class 1 compared to Class 2 [χ^2^(1) = 4.51, *p* = .034, *ω* = .05]. Intergroup contact decreased in Class 4 but was stable in Class 1 [χ^2^(1) = 5.84, *p* = .016, *ω* = .05], and Class 2 [χ^2^(1) = 7.10, *p* = .008, *ω* = .06]. Internalizing problems were higher in Class 3 compared to Class 2 at *p* ≤ .10 [χ^2^(1) = 3.48, *p* = .062, *ω* = .04], and Class 4 [χ^2^(1) = 7.20, *p* = .007, *ω* = .06], and internalizing problems were higher in Class 1 compared to Class 4 [χ^2^(1) = 5.90, *p* = .015, *ω* = .05]. Internalizing problems decreased more strongly in Class 3 compared to Class 1 at *p* ≤ .10 [χ^2^(1) = 3.70, *p* = .054, *ω* = .04], Class 2 [χ^2^(1) = 3.92, *p* = .048, *ω* = .04], and Class 4 [χ^2^(1) = 5.28, *p* = .022, *ω* = .05]. Life satisfaction was higher in Class 2 compared to Class 1 [χ^2^(1) = 6.13, *p* = .013, *ω* = .05], Class 3 at *p* ≤ .10 [χ^2^(1) = 3.33, *p* = .068, *ω* = .04]. Life satisfaction increased more strongly in Class 3 compared to Class 1 [χ^2^(1) = 9.85, *p* = .002, *ω* = .07], Class 2 [χ^2^(1) = 10.69, *p* = .001, *ω* = .07], and Class 4 [χ^2^(1) = 24.60, *p* < .001, *ω* = .11], and life satisfaction increased more strongly in Class 1 compared to Class 2 at *p* ≤ .10 [χ^2^(1) = 3.65, *p* = .056, *ω* = .04]. Health was higher in Class 4 compared to Class 3 at *p* ≤ .10 [χ^2^(1) = 3.01, *p* = .083, *ω* = .04], and increased more strongly in Class 3 compared to Class 2 at *p* ≤ .10 [χ^2^(1) = 2.84, *p* = .092, *ω* = .04], and Class 4 at *p* ≤ .10 [χ^2^(1) = 2.77, *p* = .096, *ω* = .04].

* *p* < .05, ** *p* < .01, *** *p* < .001.

*Table 11.* Model fit statistics for unconstrained multiple group models with gender, age, generational status, parent education, country of settlement, and region of origin as covariates

|  | χ^2^(df) | *p* for χ^2^ | TLI | CFI | RMSEA | 90% C.I. |
| --- | --- | --- | --- | --- | --- | --- |
| Problem behaviour at school | 60.66 (55) | .279 | 0.981 | 0.993 | .014 | .000, .032 |
| Delinquent behaviour | 65.55 (51) | .083 | 0.906 | 0.969 | .023 | .000, .038 |
| Intergroup contact | 76.20 (54) | .025 | 0.926 | 0.974 | .028 | .010, .042 |
| Internalizing problems | 78.37 (51) | .008 | 0.927 | 0.974 | .032 | .017, .045 |
| Life satisfaction | 62.30 (48) | .080 | 0.914 | 0.973 | .024 | .000, .039 |
| Health | 28.75 (54) | .998 | 1.000 | 1.068 | .000 | .000, .000 |

*Notes.* To improve the model fit for delinquent behaviour, the T1 manifest variable correlated with the Netherlands dummy. For intergroup contact and health, we freely estimated the intercepts of the T2 manifest variables. For internalizing problems, we fixed the residual variance of the T3 (instead of T1) manifest variable at 0. For life satisfaction, the residual variance of the T1 manifest variable was freely estimated, and father university degree correlated with T3 life satisfaction. Covariates include gender, age, generational status, university degree mother, university degree father, country of settlement (Germany as reference category), and region of origin (Turkey as reference category). Covariates were allowed to correlate.

*Table 12*. Results for multiple group comparisons with gender, age, generational status, parent education, country of settlement, and region of origin as covariates

|  |  | Class 1 | Class 2 | Class 3 | Class 4 |
| --- | --- | --- | --- | --- | --- |
| Problem behaviour at school | Intercept | 1.83 (.09) _a_ | 1.90 (.09) _a_ | 2.09 (.19) _a_ | 2.04 (.13) _a_ |
|  | Slope | 0.07 (.11) _a_ | 0.09 (.11) _a_ | -0.26 (.21) _a_ | -0.08 (.20) _a_ |
| Delinquent behaviour | Intercept | 0.68 (.11) _a_ | 0.71 (.09) _a_ | 0.38 (.21) _a_ | 0.94 (.16) _a_ |
|  | Slope | -0.05 (.16) _a_ | -0.24 (.14) _a_ | 0.14 (.21) _a_ | -0.22 (.18) _a_ |
| Intergroup contact | Intercept | 2.83 (.09) _a_ | 2.82 (.10) _a_ | 3.08 (.26) _a_ | 2.82 (.16) _a_ |
|  | Slope | 0.03 (.11) _a_ | 0.01 (.13) _a,b_ | 0.05 (.32) _a,b_ | -0.39 (.17)* _b_ |
| Internalizing problems | Intercept | 1.59 (.07) _a,b_ | 1.56 (.06) _a_ | 1.91 (.17) _b_ | 1.30 (.11) _c_ |
|  | Slope | -0.18 (.12) _a_ | -0.20 (.12) _a_ | -0.73 (.25) ** _b_ | -0.05 (.17) _a_ |
| Life satisfaction | Intercept | 8.36 (.23) _a,b_ | 8.84 (.06) _b_ | 7.53 (.65) _a_ | 8.88 (.37) _a,b_ |
|  | Slope | 0.26 (.16) _a_ | -0.08 (.22) _a_ | 1.53 (.50) ** _b_ | -0.21 (.32) _a_ |
| Health | Intercept | 4.03 (.10) _a_ | 4.12 (.11) _a_ | 3.65 (.25) _a_ | 4.24 (.18) _a_ |
|  | Slope | 0.38 (.13) ** _a,b_ | 0.33 (.13) ** _a,b_ | 0.82 (.27) ** _a_ | 0.21 (.18) _b_ |

*Notes.* Unstandardized effects (standard errors in parentheses). χ^2^ difference tests (*df = 1, p* ≤ .05) were conducted for each pair of classes and adjusted using the Satorra-Bentler scaling correction. Different subscripts in a row indicate differences between classes at *p* ≤ *.05*. Covariates are listed under Table 11. There were no differences between the classes in terms of problem behaviour at school. Delinquent behaviour was higher in Class 4 compared to Class 3 at *p* ≤ .10 [χ^2^(1) = 3.26, *p* = .071, *ω* = .04]. The decrease in intergroup contact in Class 4 differed from the stable levels of contact in Class 1 [χ^2^(1) = 4.11, *p* = .043, *ω* = .04], and at *p* ≤ .10 from Class 2 [χ^2^(1) = 3.36, *p* = .067, *ω* = .04]. Internalizing problems were higher in Class 3 compared to Class 2 [χ^2^ (1) = 3.97, *p* = .046, *ω* = .04], and Class 4 [χ^2^ (1) = 9.40, *p* = .002, *ω* = .07], and lower in Class 4 compared to Class 1 [χ^2^ (1) = 5.01, *p* = .025, *ω* = .05], and Class 2 [χ^2^ (1) = 3.97, *p* = .046, *ω* = .04]. Additionally, internalizing problems decreased more strongly in Class 3 compared to Class 1 [χ^2^ (1) = 4.42, *p* = .036, *ω* = .05], Class 2 [χ^2^ (1) = 4.77, *p* = .029, *ω* = .05], and Class 4 [χ^2^ (1) = 5.84, *p* = .016, *ω* = .05]. Life satisfaction was higher in Class 2 compared to Class 3 [χ^2^ (1) = 4.17, *p* = .041, *ω* = .04]. The difference in life satisfaction between Class 3 and 4 was significant at *p* ≤ .10 [χ^2^ (1) = 3.51, *p* = .061, *ω* = .04]. Life satisfaction increased more strongly in Class 3 compared to Class 1 [χ^2^ (1) = 12.45, *p* < .001, *ω* = .08], Class 2 [χ^2^ (1) = 29.13, *p* < .001, *ω* = .12], and Class 3 [χ^2^ (1) = 19.29, *p* < .001, *ω* = .10]. Health was lower in Class 3 compared to Class 2 and 4 at *p* ≤ .10 [χ^2^(1) = 3.40, *p* = .065, *ω* = .04, and χ^2^(1) = 3.80, *p* = .051, *ω* = .04, respectively], and health increased more strongly in Class 3 compared to Class 4 [χ^2^(1) = 4.27, *p* = .039, *ω* = .05], and Class 2 at *p* ≤ .10 [χ^2^(1) = 3.49, *p* = .062, *ω* = .04].

* *p* < .05, ** *p* < .01, *** *p* < .001.
